# Declarative memory profiles in children with non-specific intellectual disability: a cluster analysis approach

**DOI:** 10.3389/fpsyt.2025.1581144

**Published:** 2025-05-08

**Authors:** Urszula Sajewicz-Radtke, Ariadna Łada-Maśko, Michał Olech, Krzysztof J. Leoniak, Bartosz M. Radtke

**Affiliations:** ^1^ Laboratory of Psychological and Educational Tests, Gdańsk, Poland; ^2^ Institute of Psychology, University of Gdańsk, Gdańsk, Poland; ^3^ Department of Psychology, Medical University of Gdansk, Gdansk, Poland; ^4^ Institute of Psychology, Maria Curie-Skłodowska University, Lublin, Poland

**Keywords:** intellectual disability, declarative memory, memory profiles, cognitive functioning, children neuropsychological assessment

## Abstract

**Introduction:**

Intellectual disability (ID) is increasingly being understood as a multidimensional condition that requires assessment beyond general intelligence. While traditional approaches focus on IQ, memory impairment plays a critical role in learning and adaptation. This study explored the declarative memory profiles of individuals diagnosed with non-specific intellectual disability (NSID) to identify cognitive patterns that may inform intervention strategies.

**Methods:**

The study included 114 individuals (56 girls and 58 boys) aged 10–17 years with a confirmed mild NSID diagnosis. The participants underwent a comprehensive declarative memory assessment using the Polish version of the Test of Memory and Learning, Second Edition (TOMAL-2). A subset of 68 participants was assessed using the Stanford-Binet Intelligence Scales, Fifth Edition (SB5). Cluster analyses were performed to identify memory profiles based on the TOMAL-2 indices and their relationships with intelligence measures.

**Results:**

Two distinct memory profiles were identified. Cluster 1 (53% of the sample) exhibited lower scores across all memory indices, particularly for free recall, associative recall, and learning efficiency. Cluster 2 (47%) demonstrated relatively preserved memory abilities. Further analysis incorporating IQ measures showed that nonverbal intelligence was more strongly associated with memory performance than verbal intelligence. Notably, learning efficiency, rather than delayed verbal recall, was the strongest differentiator between the clusters.

**Conclusions:**

These findings highlight the heterogeneity of memory abilities in NSID, emphasizing the need for cognitive profiling beyond IQ. Learning potential may be a more predictive factor of functional outcomes, warranting further research and targeted interventions to enhance the adaptive capabilities in this population.

## 1 Introduction

Diagnosing cognitive functioning in individuals with intellectual disability (ID) requires a broader approach, necessitated by the introduction of new classifications, such as the International Classification of Diseases 11th Revision (ICD-11; 1) and the Diagnostic and Statistical Manual of Mental Disorders, Fifth Edition (DSM–5; 2). These classifications emphasize that intelligence quotient (IQ) alone is insufficient to capture the full scope of cognitive abilities, as it overlooks critical aspects of learning and daily adaptation. A comprehensive assessment should prioritize the evaluation of adaptive behavior, which reflects an individual’s ability to function in real-world contexts, along with a detailed analysis of cognitive functions beyond general intelligence (3).

Bertelli et al. (4) critically examined the limitations of IQ-based classification of ID and advocated a multidimensional approach incorporating specific cognitive functions. Their review highlighted that individuals with the same IQ often display distinct cognitive profiles influenced by biopsychological and neurodevelopmental factors. Traditional intelligence tests fail to capture these nuances as they primarily assess overall intelligence rather than variability in executive and cognitive functions. Neuroimaging and genetic studies support the idea that ID impairments are more closely linked to specific cognitive deficits than to a uniform reduction in general intelligence (4, 5). This perspective aligns with recent efforts to redefine ID in international classification systems, promoting a more inclusive and functionally relevant conceptualization of cognitive impairment (4, 6).

Memory and learning are fundamental cognitive processes that enable individuals to navigate social environments, acquire new skills, and achieve greater independence (7). Memory impairments can significantly affect the ability of individuals with ID to adapt to everyday challenges and engage in meaningful social and educational experiences (8). While traditional assessments of cognitive function in ID have primarily focused on general intelligence, growing evidence suggests that memory-related deficits play a crucial role in shaping functional outcomes (3, 4, 9). Understanding the specific memory profiles of individuals with ID is essential to design targeted interventions that enhance their learning potential and adaptive capabilities.

Individuals with ID commonly exhibit impairments in explicit memory processes that affect their ability to efficiently encode, store, and retrieve new information. These deficits are particularly pronounced in verbal and nonverbal tasks, with research indicating that children with ID struggle more with recall than with recognition (8, 10, 11). Furthermore, studies have shown that individuals with ID face challenges in using semantic encoding strategies, meaning they do not benefit from related word associations to enhance their memory performance. This suggests that memory impairments in ID are not solely due to reduced cognitive capacity, but also to underlying deficits in memory processing strategies, which contribute to difficulties in acquiring and retaining new knowledge (11, 12).

Research on memory function in individuals with ID has consistently demonstrated that memory impairment is neither uniform nor equally distributed across ID subtypes (13). Memory deficits vary depending on the underlying etiology, developmental trajectory, and cognitive profile of the individual. While numerous studies have examined the distinct memory profiles of well-defined syndromes, such as Down syndrome (DS; 13, 14) and Williams syndrome (WS; 10, 15), considerably fewer studies have focused on individuals diagnosed with non-specific intellectual disability (NSID; 13). NSID is characterized by impairments without a clearly defined genetic or neurodevelopmental origin, and emerging evidence indicates that memory deficits in this group can be heterogeneous, impacting short-term memory (STM), working memory (WM), and long-term memory to varying degrees (13). Given the fundamental role of memory in daily functioning, education, and adaptive skills, our study specifically focused on NSID, addressing this significant gap in the literature. A deeper understanding of the declarative memory profiles in individuals with NSID is essential for developing tailored interventions that meet their unique cognitive challenges.

Declarative memory, which includes episodic and semantic memory, plays a crucial role in learning new information, recalling past experiences, and forming knowledge structures that support academic and functional skills (16). Individuals with NSID often exhibit difficulties in verbal and visual-spatial declarative memory tasks, which can hinder their ability to effectively encode, store, and retrieve information. In contrast, some studies suggest that implicit memory processes such as procedural learning and repetition priming may be relatively preserved in individuals with ID, allowing them to acquire certain skills through repeated exposure rather than conscious recollection (8, 10, 13). However, the extent to which implicit memory remains intact in NSID is unclear, as variability in memory performance is likely to be influenced by individual differences in executive functioning, attentional control, and cognitive flexibility. Previous studies have primarily relied on small sample sizes and experimental tasks, making it difficult to develop consistent models and memory profiles for this population. By utilizing a standardized and norm-referenced assessment tool, this study allowed for the delineation of declarative memory profiles in individuals with NSID, providing a more comprehensive understanding of their cognitive strengths and weaknesses.

This study addressed two key research questions. The first is whether there are specific declarative memory profiles in individuals with NSID, given the heterogeneity observed in prior studies. Identifying distinct patterns of memory functioning could provide valuable insights into the cognitive architecture of NSID and inform individualized intervention strategies. Second, we aimed to verify whether individuals with NSID exhibit lower performance on the Verbal Delayed Recall Index, as suggested by previous findings in a broader ID population (8, 13). Given that delayed verbal recall is a critical component of declarative memory and plays a fundamental role in academic and adaptive functioning, examining this aspect in a standardized manner would contribute to a more nuanced understanding of the memory deficits in NSID. By addressing these questions, our study sought to refine existing models of memory functioning in ID and offer empirical evidence that can guide future research and clinical practice.

## 2 Materials and methods

This study was approved by The Ethics Committee for Research Projects at the Faculty of Social Sciences, University of Gdansk, Poland (decision no. 13/2022).

### 2.1 Participants and procedure

The total sample consisted of 114 individuals (56 girls and 58 boys) aged 10;00 to 17;11 years, (*M_age_* = 13.39; *SD_age_* = 1.74) with an official mild ID diagnosis (i.e., based on a completed ID diagnostic process at state centers specializing in these assessments). Inclusion criteria required a current diagnosis of non-specific intellectual disability, and the sample also included children who had additional neurodevelopmental disorders. Exclusion criteria encompassed diagnoses of genetic syndromes (such as Down syndrome, Williams syndrome, fragile X syndrome, and others), metabolic diseases, neurological disorders, or visual and hearing impairments. Additionally, individuals whose intellectual disability was not present from birth but resulted from illness, injury, or damage occurring later in life were also excluded. Individual participants (9%) were also diagnosed with developmental disorders, speech development disorders, or ADHD. All the participants were enrolled in a special education program, either in mainstream or special schools, across different places of residence. Most participants’ parents (93% of mothers and 92% of fathers) were not educated at the tertiary level. All participants underwent complete declarative memory assessment using the Polish version of the Test of Memory and Learning, Second Edition (TOMAL-2; 17), while a randomly selected subsample of 68 participants (*M_age_* = 13.35; *SD_age_* = 1.82) was additionally administered the Stanford Binet Intelligence Scale–Fifth Edition (SB5; 18). **Table 1** presents the total sample composition, including the subsample of participants who were administered both study measures (i.e., TOMAL-2 and SB5).

**Table 1:** Demographic characteristics of the study sample.

Variable	Total (*N* = 114)	Subsample (*n* = 68)
	*n* [%]	*n* [%]
Sex		
Female	56 [49]	33 [48]
Male	58 [51]	35 [52]
Age Group		
10;00–11;11	30 [26]	19 [28]
12;00–13;11	37 [33]	22 [32]
14;00-15;11	36 [32]	19 [28]
16;00-17;11	11 [9]	8 [12]
Additional Diagnosis *(more than one possible)*		
Developmental Disorders	4 [4]	1 [1]
ADHD	1 [1]	1 [1]
Speech Development Disorders	5 [4]	4 [6]
Educational Institution		
Primary School	67 [59]	53 [78]
Special Education School	47 [41]	15 [22]
Size of Place of Residence		
Less than 5,000 residents	20 [18]	15 [22]
Between 5,000 and 100,000 residents	42 [37]	34 [50]
More than 100,000 residents	52 [46]	19 [28]
Mother’s Education		
Primary	19 [17]	14 [21]
Vocational	54 [47]	34 [50]
Secondary	33 [29]	17 [25]
Bachelor’s/Engineering	2 [2]	1 [1]
Master’s Degree	1 [1]	–
Missing data	5 [4]	2 [3]
Father’s Education		
Primary	23 [20]	14 [21]
Vocational	55 [48]	37 [54]
Secondary	27 [24]	14 [21]
Bachelor’s/Engineering	–	–
Master’s Degree	2 [2]	–
Missing data	7 [6]	3 [4]

### 2.2 Procedure

All assessments were conducted by qualified diagnosticians from psychological and pedagogical counseling centers in Poland from 2022 to 2024. Psychological and educational counseling centers across Poland received information on the study. Counseling center psychologists interested in participating in this study received training on the research protocol. Psychologists enlisted children from schools and other educational institutions. Parents were informed by a psychologist that the counseling center was taking part in scientific research and about the scope of the study and the data provided. All parents of children who participated in the study provided written consent for participation. Sensitive personal information was not obtained. No compensation was received for this study.

### 2.3 Measures

A comprehensive evaluation of memory functions (including free and associative recall, meaningful and abstract memory, sequential recall, and learning) was performed using the Polish version of the standardized memory battery TOMAL-2 (17). The core battery comprises eight subtests divided into four verbal and four nonverbal tasks used to calculate verbal and nonverbal memory indices, respectively. In addition, the TOMAL-2 includes four verbal and two nonverbal supplementary subtests designed to offer additional, more detailed memory indices: the Verbal Delayed Recall Index (VDRI), Attention/Concentration Index (ACI), Sequential Recall Index (SRI), Free Recall Index (FRI), Associative Recall Index (ARI), and Learning Index (LI). The reliability of the individual indices of the TOMAL-2 test is also high to very high. For the core indexes, Cronbach’s alpha reaches values between 0.94 and 0.96, while for the supplementary indexes, it falls within the range of 0.87 to 0.95 (17).

Intelligence was measured using the Polish version of the Stanford Binet Intelligence Scale–Fifth Edition (SB5; 18). The SB5 is widely used as a specialized individual test to assess intelligence, particularly in special needs groups. The full IQ scale consists of 10 subscales—referring to the five cognitive factors (i.e., fluid reasoning, knowledge, quantitative reasoning, visual spatial processing, and working memory)—used to calculate general Nonverbal Intelligence (five subscales) and Verbal Intelligence (five subscales) quotients. The reliability of the main indexes of the SB5 (full scale IQ, nonverbal IQ, verbal IQ), measured by the Spearman-Brown inter-rater equivalence coefficients, is very high, ranging from 0.95 to 0.98. When measured using Cronbach’s alpha for internal consistency, it falls within the range of 0.93 to 0.96 (18).

### 2.4 Statistical analysis

To address the research questions, two independent cluster analyses were performed using 1) six supplementary memory indices from the TOMAL-2 test (VDRI, ACI, SRI, FRI, ARI, and LI) that were assessed in the total sample (*N* = 114), and 2) two general memory indices from the TOMAL-2 test (Verbal Memory, Nonverbal Memory) and two general IQ indices from SB5 (Verbal IQ, Nonverbal IQ) that were assessed in the subsample (*n* = 68).

Cluster analysis followed an identical protocol. First, the number of clusters was determined using the NbClust function in the R NbClust package (19). This function checks 30 criteria (including the commonly deployed silhouette method and gap statistics) to determine the number of clusters in the dataset. Next, the *k*-means clustering method was used to fit the cluster models to the determined number of clusters (20). The Hartigan and Wong algorithm was used to minimize the Euclidean distances of all points to their nearest cluster centers by minimizing the within-cluster sum of squared errors (21). After fitting the models, independent sample *t*-tests (performed after controlling for the normality assumption of the data with density plots) with Cohen’s *d* effect size calculations were performed to analyze the profiles of the results (based on the chosen indices) assigned to the established clusters, thus providing insights into the magnitude of group differences. Simultaneously, chi-square analyses were used to determine whether participants’ demographic characteristics were associated with their membership in each cluster. All analyses were conducted in the R environment using R Studio software (22).

## 3 Results

### 3.1 Cluster analysis of memory indices

**Figure 1:**
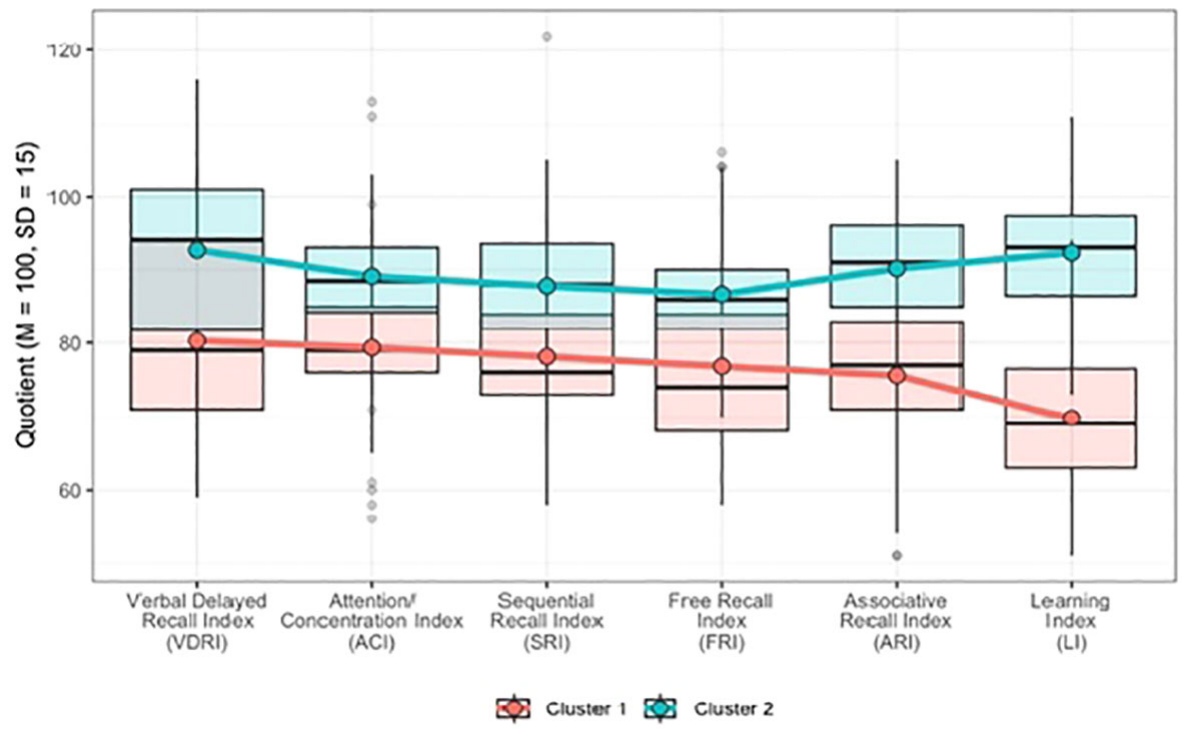
Distribution of standardized scores from the six supplementary indices of the TOMAL-2 test across clusters.

Testing for the optimal number of clusters revealed that in terms of specific memory indices, the study sample (*N* = 114) was best described by two clusters. The *k*-means clustering method accounted for 53% of the sample in Cluster 1 and 47% in Cluster 2. **Figure 1** shows the distribution of the standardized TOMAL-2 scores (six supplementary indices) within the cluster profiles. **Table 2** summarizes the values of the cluster centers and the differences across all six indices among the distinguished groups.

**Table 2:** Cluster comparisons across the six supplementary TOMAL-2 indices.

Variable	Cluster 1 (*n* = 60)		Cluster 2 (*n* = 54)		*t*	*p*	*d* [95% CI]
	*M*	*SD*	*M*	*SD*			
Verbal Delayed Recall Index	80.28	13.73	92.65	11.31	–5.27	<.001	0.99 [0.59; 1.37]
Attention/Concentration Index	79.37	8.76	89.13	8.19	–6.15	<.001	1.15 [0.75; 1.55]
Sequential Recall Index	78.15	8.62	87.81	9.49	–5.67	<.001	1.06 [0.67; 1.46]
Free Recall Index	76.83	10.39	86.70	7.80	–5.77	<.001	1.08 [0.67; 1.46]
Associative Recall Index	75.60	10.14	90.17	8.56	–8.31	<.001	1.56 [1.13; 1.96]
Learning Index	69.82	9.70	92.29	7.38	–13.99	<.001	2.63 [2.10; 3.10]

Chi-square analyses revealed no statistically significant association between cluster membership and participants’ demographic characteristics, such as sex [*χ* ^2^ (1, N = 114) = 0.86, *p* = .46], age group [*χ* ^2^ (3, N = 114) = 0.05, *p* = .99], or educational institution [*χ* ^2^ (1, N = 114) = 0.01, *p* = .92]. Importantly, independent sample t-tests (**Table 2**) revealed statistically significant differences across all six indices of memory performance between established clusters (t-values ranged from –5.27 to –13.99, all *p* <.001). The effect sizes (Cohen’s *d*) ranged from 0.99 (Verbal Delayed Recall Index) to 2.63 (Learning Index), indicating large effects for these differences. Taken together, the participants from Cluster 1 exhibited lower scores across all indices, whereas those from Cluster 2 consistently displayed higher scores for all indices.

### 3.2 Cluster analysis of memory indices and intelligence quotients

**Figure 2:**
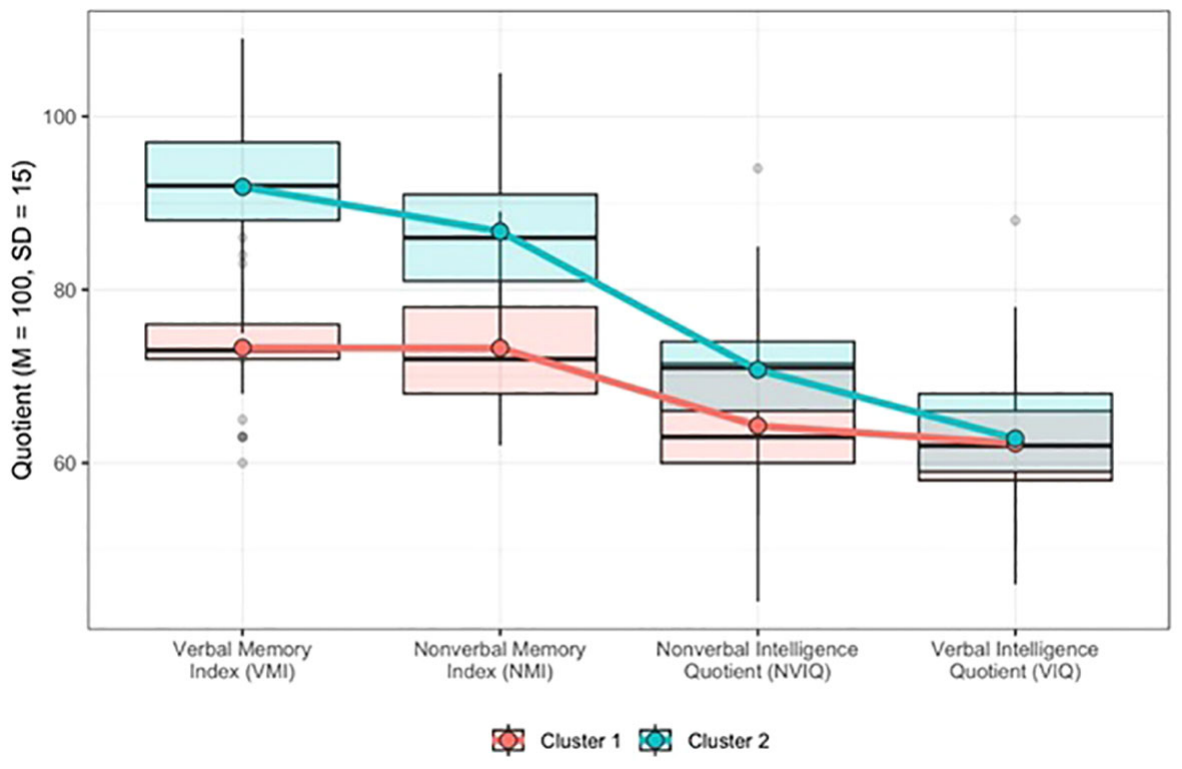
Distribution of standardized TOMAL-2 test and SB5 test scores between two identified clusters.

When considering general memory indices (Verbal Memory Index, Nonverbal Memory Index) and IQ (Nonverbal IQ, Verbal IQ), testing for the optimal number of clusters revealed that the subsample (*n* = 68) was best described by two clusters. The *k*-means clustering method accounted for 46% of the sample in Cluster 1 and 54% in Cluster 2. **Figure 2** shows the distribution of the standardized scores from the two general memory indices (TOMAL-2 test) and two general IQ indices (SB5) within the identified cluster profiles. **Table 3** summarizes the values of the cluster centers and the differences across all four indices between the distinguished groups.

**Table 3:** Cluster comparisons across verbal and nonverbal memory and IQ indices.

Variable	Cluster 1 (*n* = 31)		Cluster 2 (*n* = 37)		*t*	*p*	*d* [95% CI]
	*M*	*SD*	*M*	*SD*			
Verbal Memory Index	73.29	6.19	91.86	8.84	–10.15	<.001	2.47 [1.77; 3.02]
Nonverbal Memory Index	73.26	7.01	86.73	7.31	–7.74	<.001	1.88 [1.30; 2.45]
Nonverbal Intelligence Quotient	64.29	8.32	70.76	7.79	–3.29	<.01	0.80 [0.31; 1.30]
Verbal Intelligence Quotient	62.26	7.51	62.81	8.21	–0.29	.77	0.07 [-0.41; 0.55]

Supplementary chi-square analyses revealed no statistically significant association between cluster membership and participants’ demographic characteristics, such as sex [*χ* ^2^ (1, N = 68) = 0.001, *p* = .98], age group [*χ* ^2^ (3, N = 68) = 2.34, *p* = .50], or educational institution [*χ* ^2^ (1, N = 68) = 0.24, *p* = .62]. However, independent sample t-tests (**Table 3**) revealed statistically significant differences across three of the four indices between established clusters (t-values ranged from –3.29 to –10.15, all *p* <.001). While participants from both clusters differed significantly in terms of general verbal and nonverbal memory indices, the only significant difference in IQ was observed on the nonverbal scale. The effect sizes (Cohen’s *d*) ranged from 0.80 (Nonverbal IQ) to 2.63 (Verbal Memory Index), indicating large effects of these differences.

## 4 Discussion

This study identified two distinct declarative memory profiles in the NSID group. Cluster analysis revealed that 53% of the participants belonged to Cluster 1, which was characterized by significantly lower memory performance across all assessed indices, whereas 47% belonged to Cluster 2, demonstrating consistently higher memory scores. Cluster 1 individuals exhibited substantial difficulties in verbal and nonverbal memory with pronounced deficits in free recall (FRI), associative recall (ARI), and learning (LI). In contrast, Cluster 2 participants showed relatively preserved memory function, particularly in tasks requiring sequential recall (SRI) and attention/concentration (ACI). The existence of these two profiles highlights the heterogeneity of memory abilities in NSID and underscores the need for tailored cognitive interventions based on individual memory strengths and weaknesses.

The application of cluster analysis was motivated by the need to identify distinct cognitive profiles in children with NSID, particularly in declarative memory. Traditional methods often assume homogeneity within diagnostic categories, overlooking meaningful variations (4). Given the diversity of memory functioning, a data-driven approach like cluster analysis provides a clearer picture of cognitive heterogeneity (3). Declarative memory, essential for learning and adaptation, varies significantly among individuals with NSID (16). Cluster analysis identifies natural groupings without predefined categories, making it well-suited for exploratory studies in cognitive profiling (12). Similar approaches have been used in neurodevelopmental research to uncover cognitive subtypes (23). Hierarchical clustering, while useful, is computationally intensive and prioritizes pairwise similarities over representative cluster centers. Factor analysis identifies latent dimensions but does not categorize individuals into profiles. Supervised classification methods require predefined categories, limiting exploratory insights. K-means clustering, used in this study, provides interpretable profiles while minimizing within-group variance (21). Our findings emphasize the need for individualized interventions. Children with severe recall deficits may benefit from structured repetition, while those with relatively preserved memory require support in executive function and attentional control (3). The strong link between nonverbal intelligence and memory performance highlights the utility of visuospatial learning techniques (23). Cluster analysis provided a refined understanding of declarative memory heterogeneity in NSID.

Importantly, no significant sex differences were found in cluster membership, allowing for a broader generalization of our findings across male and female individuals with NSID. This suggests that the observed variability in declarative memory functioning is primarily driven by individual cognitive differences rather than demographic influences. This aligns with previous research suggesting that cognitive impairment in individuals with ID may not be strongly influenced by gender, reinforcing the need for comprehensive cognitive profiling beyond demographic variables (8).

Although demographic factors did not play a significant role in differentiating memory profiles in our sample, future studies should explore potential moderating variables such as socio-economic status, educational access, and comorbid conditions (e.g., ADHD, language impairments). These factors may influence cognitive functioning in ways that were not captured in the present study. Understanding these influences could further refine intervention strategies, ensuring they address the broader context of cognitive development in children with NSID.

Our second research question addressed whether the Verbal Delayed Recall Index (VDRI) was the most distinguishing factor between the two clusters, as suggested by previous studies comparing individuals with ID to their typically developing peers (8, 13). Contrary to our hypothesis, the VDRI differences between clusters were relatively moderate (*d* = 0.99), and the Learning Index (LI) exhibited the largest effect size (*d* = 2.63), making it the strongest differentiator between the two groups. This finding has significant implications for cognitive assessment and intervention for NSID. Greenspan et al. (3) argued that IQ alone is an insufficient measure of cognitive functioning, and our results suggest that learning potential, as captured by LI, may be a predictive factor for cognitive adaptability and real-world functioning. Future studies should explore whether LI can serve as a more reliable indicator of cognitive and adaptive outcomes than traditional IQ measures.

Furthermore, when IQ was incorporated into the cluster analysis, participants in the two clusters differed significantly in both general verbal and nonverbal memory indices. However, the only significant IQ difference was observed in the Nonverbal Intelligence Quotient (NVIQ), suggesting that nonverbal intelligence may have a stronger association with memory performance than verbal intelligence in NSID. This finding highlights the potential role of visuospatial processing and nonverbal problem-solving skills in shaping memory outcomes in this population (23).

This study has several limitations that should be considered when interpreting the findings. First, the sample consists exclusively of children from Poland, which raises concerns about the generalizability of the results to other populations where access to education, intervention strategies, and support services may differ. Additionally, the sample size (*N* = 114) is relatively small, which limits the statistical power and robustness of the findings. Future research should consider several factors that may influence cognitive functioning and memory performance. These include potential confounding variables such as socioeconomic status (SES) and comorbid conditions (e.g., ADHD), which could shape declarative memory profiles in children with non-specific intellectual disability. It remains to be explored whether these factors significantly impact the observed results. Addressing these limitations in future studies may provide a more comprehensive understanding of memory functioning in this population.

Overall, the results of this study emphasize the need to move beyond traditional IQ-based assessments when evaluating cognitive functioning in NSID. While IQ provides a broad measure of intellectual ability, it does not capture the variability in specific cognitive processes that are essential for learning and adaptation. Our findings suggest that analyzing first-stratum cognitive abilities within the Cattell-Horn-Carroll theory (CHC; 24), such as learning efficiency, memory retrieval, and sequential processing, is crucial for a more accurate understanding of cognitive functioning in NSID. Future research should investigate how these lower-level cognitive processes contribute to adaptive behavior and everyday problem-solving in individuals with NSID, ultimately providing more effective support strategies and educational interventions.

Given that learning efficiency emerged as the strongest differentiator between memory profiles, intervention strategies should focus on structured, repetition-based learning and scaffolded instruction. Techniques such as visual schedules, step-by-step task breakdowns, and semantic clustering can help reinforce memory retention in children with non-specific intellectual disability. Additionally, the strong link between nonverbal intelligence and memory performance suggests that visuospatial learning methods—including pictograms, diagrams, and hands-on activities—should be integrated into educational programs.

Furthermore, targeted attention and concentration training, such as pattern recognition games and cognitive exercises, could enhance focus and improve overall memory function. Classroom accommodations, including extended time for recall tasks, interactive retrieval sessions, and the use of recorded lessons, may also help compensate for memory-related difficulties and improve learning outcomes.

Future research should examine the effectiveness of these strategies in practice to refine intervention approaches further and ensure they are tailored to the individual cognitive profiles of children with NSID. Expanding diagnostic frameworks and engaging multidisciplinary teams will enhance our understanding of this group’s unique cognitive functioning, ultimately leading to more effective educational planning and therapeutic interventions that better prepare them for daily life.

## Data Availability

The raw data supporting the conclusions of this article will be made available by the authors, without undue reservation.
